# Hybrid Fractional Laser and Autologous Lipofilling: A Synergistic Strategy for Functional and Aesthetic Scar Remodeling

**DOI:** 10.3390/jcm14196708

**Published:** 2025-09-23

**Authors:** Gabriele Delia, Lucia Quattrocchi, Pietro Micieli, Damiano Tambasco, Roberta Albanese, Fabiana Battaglia

**Affiliations:** 1 Department of Plastic and Reconstructive Surgery, University Hospital of Messina “AOU Gaetano Martino”, 98125 Messina, Italy; gabrieledelia@gmail.com (G.D.); quattrocchilucia@outlook.it (L.Q.); petrus.mic@gmail.com (P.M.); 2 Plastic Surgery Unit, Ospedale San Carlo di Nancy, Via Aurelia 275, 00165 Rome, Italy; damianotambasco@gmail.com (D.T.); albaneseroberta16@gmail.com (R.A.)

**Keywords:** scar treatment, lipofilling, hybrid laser, regenerative medicine, ADSCs, CO_2_ laser, tissue remodeling

## Abstract

**Background:** Scar management remains a significant challenge in plastic and reconstructive surgery, particularly when addressing atrophic, retractile, or fibrotic scars. Autologous fat grafting and hybrid fractional laser therapy have independently shown efficacy in improving scar quality. This study aims to evaluate the synergistic effect of their combination on clinical and functional scar outcomes. **Methods:** A prospective, comparative study was conducted on patients with cutaneous scars of various etiologies. Participants were treated with either hybrid fractional laser therapy alone (CO_2_ and 1570 nm Erbium-glass wavelengths) or a combined protocol of laser plus autologous lipofilling. Clinical outcomes were assessed at baseline and at 30, 60, and 90 days post-treatment using the Vancouver Scar Scale (VSS), patient satisfaction scores, and Visual Analog Scale (VAS) for pain and discomfort. **Results:** Patients receiving the combined treatment demonstrated significantly greater improvement in scar pigmentation, elasticity, pliability, and thickness compared to those treated with laser alone. Subjective symptoms, including pain and itching, were also more effectively alleviated. The volumetric and regenerative properties of adipose tissue, particularly its content of adipose-derived stem cells (ADSCs) and stromal vascular fraction (SVF), likely contributed to the enhanced outcomes observed. **Conclusions:** The combination of hybrid fractional laser therapy and autologous lipofilling offers a superior therapeutic strategy for scar remodeling compared to laser monotherapy. This integrated regenerative approach addresses both structural and biological aspects of scar tissue, making it a valuable protocol for personalized and effective scar management. Further randomized trials with larger sample sizes and histological validation are warranted to confirm these preliminary findings and refine therapeutic protocols.

## 1. Introduction

Cutaneous scars represent a frequent and often underestimated sequela of trauma, surgery, or burns. While scarring is a physiological outcome of wound healing, its manifestations can result in significant aesthetic, functional, and psychological morbidity. Hypertrophic, retractile, or dyschromic scars can impair joint mobility, induce chronic pain or pruritus, and adversely affect self-image and social functioning [1].

Over the past two decades, the clinical management of scars has evolved from excisional or ablative approaches toward regenerative strategies aimed at restoring the architecture and function of damaged skin. Among these, fractional laser technology has become a cornerstone of scar therapy, promoting dermal remodeling via controlled thermal injury. Hybrid fractional lasers that combine ablative CO_2_ and non-ablative 1570 nm wavelengths have demonstrated superior efficacy in enhancing skin texture, tone, and pliability, while reducing downtime and adverse effects [2].

Concurrently, advances in autologous adipose tissue grafting have introduced new regenerative options in scar treatment. Techniques such as lipofilling and nanofat grafting allow the delivery of stromal vascular fraction (SVF) and adipose-derived stem cells (ADSCs), which play a critical role in modulating inflammation, stimulating angiogenesis, and enhancing extracellular matrix regeneration [3,4].

Lipofilling and nanofat grafting, although both derived from autologous adipose tissue, differ substantially in their characteristics and clinical purpose. Lipofilling preserves viable adipocytes and provides mainly a volumetric effect, complemented by regenerative properties from the stromal vascular fraction. Conversely, nanofat is obtained through mechanical emulsification, which disrupts mature adipocytes but retains stromal cells and adipose-derived stem cells. As a result, nanofat lacks volumetric capacity but exerts potent regenerative, angiogenic, and anti-fibrotic effects, making it suitable for intradermal applications and scar quality improvement.

The combination of fractional laser therapy with lipofilling has recently gained interest for its synergistic potential. The laser induces a pro-regenerative microenvironment, enhancing graft integration and fibroblast activation, while adipose-derived elements provide the biological stimulus for sustained tissue repair [5]. However, to date, few studies have evaluated the clinical outcomes of this combined approach in a heterogeneous population of scars.

Hybrid fractional laser therapy exerts its effects mainly through controlled photothermal stimulation, creating microscopic zones of ablation and coagulation that trigger a cascade of wound-healing responses. This process enhances fibroblast proliferation, induces neocollagenesis, reorganizes disordered collagen bundles, and promotes angiogenesis, ultimately improving scar pliability and texture [6,7,8]. In parallel, autologous fat grafting provides both structural and biological contributions to scar remodeling. Beyond its volumetric role, adipose tissue is enriched with stromal vascular fraction (SVF) and adipose-derived stem cells (ADSCs), which secrete a wide range of cytokines and growth factors such as VEGF, HGF, and IL-10. These factors stimulate angiogenesis, regulate extracellular matrix remodeling, and exert anti-fibrotic and immunomodulatory effects [9,10,11,12].The regenerative cell populations contained in SVF also interact with local fibroblasts and endothelial cells, further enhancing tissue repair and softening fibrotic scar tissue [13,14]. The combination of photothermal stimulation by hybrid fractional laser and the biological regenerative properties of autologous adipose tissue thus provides a strong mechanistic basis for their synergistic application in scar management.

Previous studies have already suggested the potential benefit of combining fractional CO_2_ laser with adipose tissue derivatives in scar treatment [2]. reported that autologous nanofat injection combined with fractional CO_2_ laser significantly improved atrophic acne scars compared to laser monotherapy. Similarly, Roohaninasab et al. [5] demonstrated superior outcomes in burn scars when fractional CO_2_ laser was combined with stromal vascular fraction. La Padula et al. [15] also confirmed that the integration of autologous fat grafting with fractional CO_2_ laser optimized both aesthetic and functional results in patients with severe facial burn scars. These findings highlight the synergistic regenerative potential of associating laser-induced dermal remodeling with adipose-derived stem cell therapy.

In this prospective study, we assessed the safety and efficacy of hybrid fractional laser therapy, alone or in combination with autologous fat grafting (via lipofilling and/or nanofat), in the treatment of scars of various etiologies and anatomical sites. We also explored the impact of treatment timing, immediate, early, or delayed, on clinical and patient-reported outcomes. Our goal was to develop an evidence-based protocol that integrates photothermal stimulation with regenerative cell therapy for optimized scar management.

## 2. Materials and Methods

**Figure 1:**
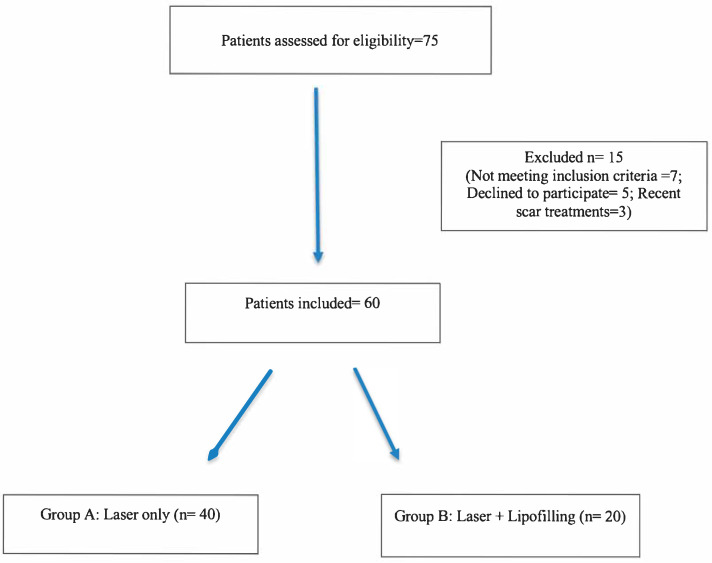
Flow diagram of patient screening and allocation into study groups. *Inclusion criteria:* patients aged 18–70 years; hypertrophic, dyschromic, or retractile scars of at least 6 months’ duration. *Exclusion criteria:* systemic diseases affecting healing, coagulopathies, active skin infections, previous scar treatments within 6 months.

This prospective comparative study involved sixty patients affected by hypertrophic, dyschromic or retractile scars of varying etiology, including post-surgical, post-traumatic and post-burn lesions. The research was conducted between 2021 and 2024 at a single center, following institutional ethical approval and written informed consent from all participants. Patients were divided into two groups (Figure 1). All participants were fully informed about the study procedures and provided written informed consent prior to enrollment. Allocation to the groups was not randomized but based on scar characteristics and patient preference after counseling. Patients with atrophic or adherent scars requiring volumetric correction were treated with combined hybrid fractional laser and lipofilling, while patients with more superficial scars or who declined fat grafting underwent laser treatment alone:

- Group A (Laser Only):
  40 patients treated exclusively with a hybrid fractional laser system combining a 10,600 nm ablative CO_2_ laser and a 1570 nm non-ablative laser. This cohort corresponds to the population previously reported in our recent publication [16].
- Group B (Laser + Lipofilling):
  20 patients underwent the same hybrid laser protocol in combination with autologous fat grafting. Each patient received 3–4 monthly laser sessions. Lipofilling was generally performed as a single procedure immediately after the first laser session. In patients with more extensive or adherent scars, a second grafting session was performed 4–6 weeks later, which we referred to as a “staged” procedure.

Inclusion criteria included patients aged 18–70 years with hypertrophic, dyschromic, or retractile scars of at least 6 months’ duration. Exclusion criteria comprised systemic diseases affecting healing, coagulopathies, active skin infections, and previous scar treatments within 6 months.

The laser therapy was administered using a device combining fractional ablative CO_2_ and non-ablative 1570 nm Erbium-glass wavelengths (Alma Lasers, Caesarea, Israel). Each session was preceded by application of a topical anesthetic cream containing lidocaine and tetracaine at 6% concentration, left in place for 40 min. A 7 × 7-pixel tip with an effective area of 1.21 cm^2^ and 300-micron spot size was employed. The CO_2_ component was delivered at 50 watts with a pulse duration between 0.8 and 1.6 milliseconds, while the Erbium-glass component operated at 8 watts with a pulse duration of 1.0 to 1.6 milliseconds. Based on scar morphology, one or two passes were performed per session, with a total of three to four monthly sessions per patient. Following treatment, a topical reparative cream was applied and strict photoprotection was recommended for all sun-exposed areas.

### Autologous Fat Harvesting and Grafting

Lipofilling: The procedure began with adipose tissue collection through liposuction, utilizing donor sites such as the abdomen, hips, or thighs based on tissue availability. A Klein solution containing adrenaline (1:200,000), local anesthetic, and physiological saline (NaCl 0.9%) was injected into the donor site. Liposuction was performed using a thin, blunt-tipped cannula with a diameter of 1.5 mm and 8 orifices of 600 nm, connected to a 10 mL syringe for manual aspiration or a suction device with a collection canister for larger amounts of adipose tissue. Subsequently, the lipoaspirate was allowed to settle for 10 min.

Nanofat processing: The obtained fat was mechanically emulsified by passing it between two 1 mm-diameter female-to-female Luer-Lock connector syringes for a total of 30 passes. Finally, the lipoconcentrate was injected subcutaneously using a blunt-tipped single-hole 0.9 mm cannula.

Grafting was performed immediately after laser treatment using retrograde injection techniques in both the subdermal and superficial dermal layers.

Clinical evaluation was performed at baseline and after 1, 2, and 3 months from the last treatment session. Standardized photographs were taken under controlled conditions for documentation and comparison. Scar quality was objectively assessed using the Vancouver Scar Scale (VSS), which measures vascularity, pigmentation, pliability and thickness.

Follow-up visits were scheduled at 30-day intervals. At each visit, patients were asked to report changes in pain, itching, or functional limitation. No patients were lost to follow-up, and compliance was monitored through telephone reminders and patient diaries.

Subjective satisfaction was recorded using a four-level scale ranging from “very satisfied” to “unsatisfied.” Pain during treatment was rated on a visual analog scale (VAS) from 0 to 10. In patients with functionally limiting scars, range of motion was evaluated before and after treatment using goniometric assessment.

Therefore, in our protocol, lipofilling was primarily employed for volume restoration in atrophic or adherent scars, while nanofat was injected intradermally in cases requiring improvement of skin quality, pigmentation, and elasticity.

## 3. Results

A total of 60 patients completed the study: 40 were treated with hybrid fractional laser therapy alone (Laser Only group), and 20 received a combined treatment of laser and autologous fat grafting (Laser + Lipofilling group). Baseline demographic and clinical characteristics are summarized in Table 1. There were no statistically significant differences between the groups in terms of age, sex distribution, scar etiology, location, or duration, confirming the initial comparability of the two cohorts.

**Figure 2:**
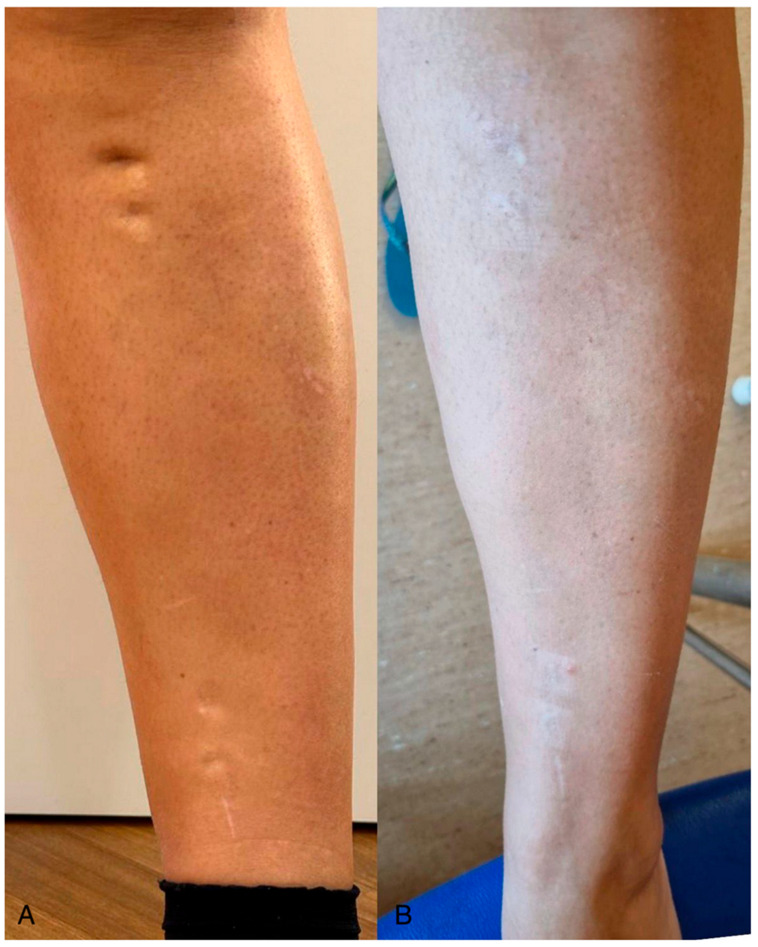
A 43-year-old female patient with a traumatic scar on the right calf. (**A**) Pre-treatment image reveals skin irregularities. (**B**) Post-treatment image (3 months after laser and lipofilling) demonstrates substantial improvement in skin tone uniformity and scar integration with surrounding tissue.

**Figure 3:**
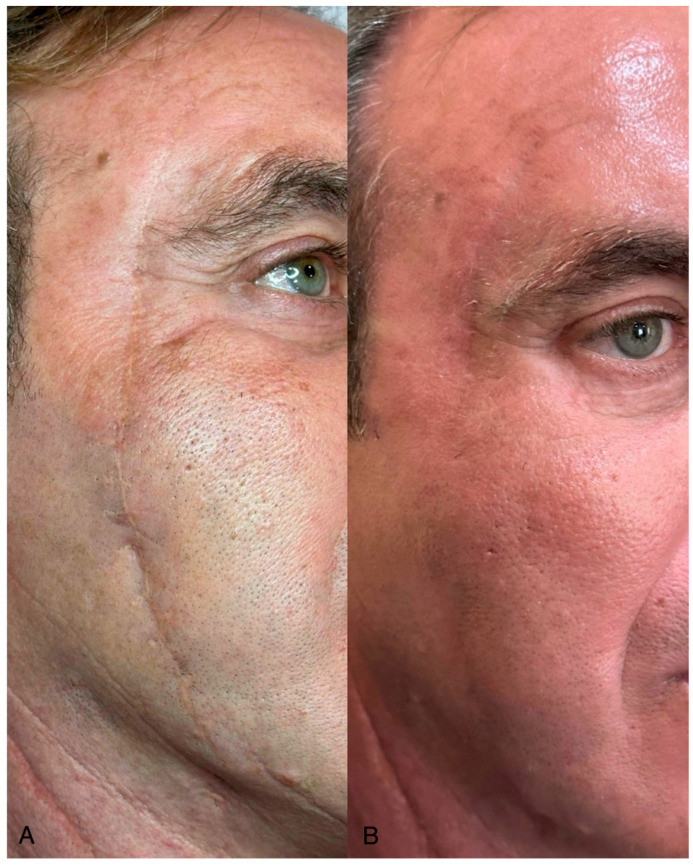
A 57-year-old male patient with a visible scar. (**A**) The image shows the scar before treatment. (**B**) The image displays the scar three months after combined hybrid fractional CO_2_ laser and autologous lipofilling, showing marked improvement in skin texture, pigmentation, and scar thickness.

Objective assessment using the Vancouver Scar Scale (VSS) showed progressive improvement in both groups over the follow-up period (T30, T60, T90). However, patients in the Laser + Lipofilling group exhibited greater overall improvement in pigmentation, pliability, and thickness (Figure 2 and Figure 3).

At day 90, mean VSS scores were significantly lower in the combined group (2.88 ± 0.92) compared to the Laser Only group (4.53 ± 1.27), although this difference did not reach statistical significance (*p* = 0.394). Although the differences between groups did not reach statistical significance, this study was likely underpowered to detect small effect sizes. Therefore, the observed trends should be interpreted with caution, while recognizing their potential clinical relevance.

Subjective symptoms were evaluated using the Visual Analog Scale (VAS) for pain and discomfort. As reported in Table 2, both groups experienced a reduction in pain levels over time; however, the Laser + Lipofilling group consistently reported lower scores at all time points. By day 90, mean VAS was 1.23 ± 0.84 for the combined group, compared to 3.61 ± 1.17 in the Laser Only group (*p* = 0.107) (Table 2).

**Figure 4:**
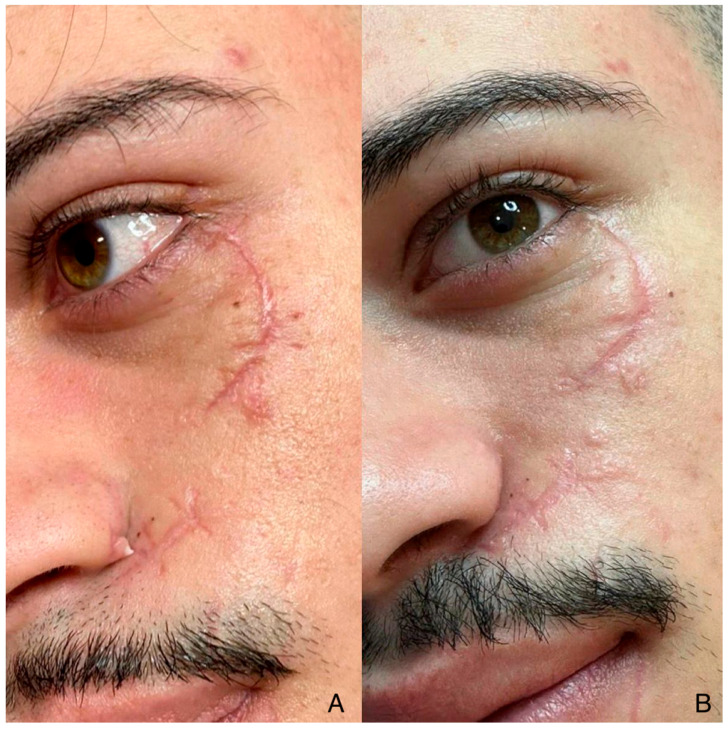
A 25-year-old male patient presented with a curved, hypertrophic post-traumatic scar extending from the infraorbital region to the mid-cheek. (**A**) The image shows the scar before treatment. (**B**) The image, taken 3 months after fractional CO_2_ laser therapy alone, demonstrates improved scar flattening, decreased erythema, and more regular skin texture, although some residual irregularities remain visible.

A representative clinical example of a patient treated with hybrid laser therapy alone is shown in Figure 4.

Table 3 summarizes key clinical variables, reported post-treatment downtime, and subjective satisfaction levels across the cohort (Table 3). These data provide a broader perspective on patient response and tolerability of the treatments, highlighting high rates of satisfaction, particularly in the combined therapy group.

Data refer to both treatment groups (Laser Only and Laser + Lipofilling), including scar types, Fitzpatrick skin types, mean downtime, and distribution of satisfaction levels. Values are expressed as absolute numbers or mean ± standard deviation.

In conclusion, although statistical significance was not achieved, likely due to the sample size, the clinical outcomes suggest that combining lipofilling with hybrid laser treatment offers superior scar remodeling and symptom relief, making it a promising strategy for integrative scar management. Although not statistically significant, the differences observed may still hold clinical relevance. The sample size was determined based on clinical feasibility, and the study may have been underpowered to detect small effect sizes. Further randomized controlled trials with larger cohorts and longer follow-up are warranted to validate these findings.

### Statistical Analysis

We conducted a comparative statistical analysis between two treatment groups: patients receiving hybrid fractional laser therapy alone (“Laser Only”) and those undergoing a combined protocol of laser plus autologous lipofilling (“Laser + Lipofilling”).

Group comparisons were performed using an unpaired two-tailed t-test. Statistical analyses were performed using SPSS version 27.0 (IBM Corp. Armonk, NY, USA). Data normality was tested using the Shapiro-Wilk test. A *p*-value < 0.05 was considered statistically significant. All analyses were conducted by the authors with methodological support from the department’s biostatistical unit.

The primary outcome measures were:

- Vancouver Scar Scale (VSS), used to assess scar severity based on vascularity, pigmentation, pliability, and height.
- Visual Analog Scale (VAS), used to quantify patient-reported pain and discomfort.

**Figure 5:**
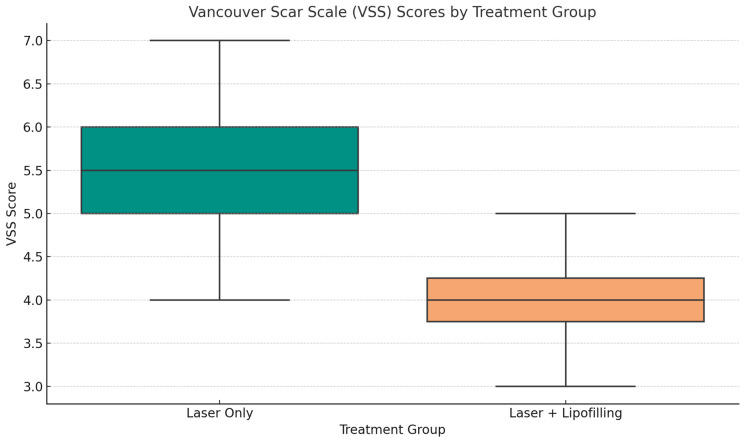
Boxplot of Vancouver Scar Scale (VSS) scores comparing “Laser Only” and “Laser + Lipofilling” treatment groups.

VSS Scores: Although the mean VSS score was lower in the Laser + Lipofilling group, indicating improved scar quality, the difference did not reach statistical significance (*p* = 0.394). As illustrated in Figure 5, patients in the Laser Only group exhibited higher median VSS values and a broader interquartile range, suggesting more severe and variable scarring. In contrast, the combined group showed narrower distribution and lower maximum values, reflecting more consistent and favorable clinical outcomes. These results support the potential regenerative effect of adipose-derived stromal components in enhancing scar remodeling.

**Figure 6:**
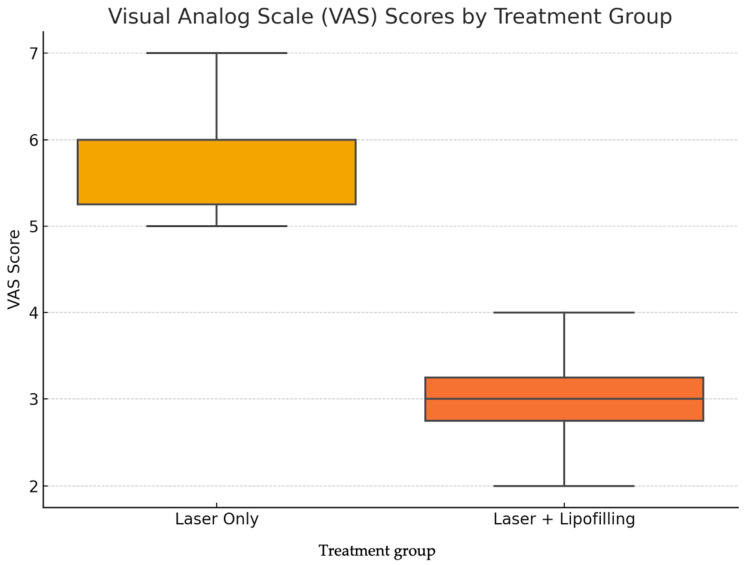
Boxplot of Visual analog scale (VAS) scores comparing “Laser Only” and “Laser + Lipofilling” treatment groups. Graphical comparisons (Figure 4 and Figure 5) illustrate the distribution of VSS and VAS scores using boxplots. These figures show a narrower interquartile range and lower maximum values in the Laser + Lipofilling group, indicating more uniform and consistent improvement across patients. These findings support the hypothesis that the regenerative effects of adipose-derived stem cells (ADSCs) and stromal vascular fraction (SVF) enhance the efficacy of fractional laser therapy.

VAS Scores: Patients treated with the combined approach also reported greater pain reduction, with a lower mean VAS score compared to the Laser Only group. However, this difference was not statistically significant (*p* = 0.107). The boxplot shown in Figure 6 illustrates the distribution of VAS scores. Patients in the Laser + Lipofilling group exhibited greater overall improvement in pigmentation, pliability, and thickness.

At day 90, mean VSS scores were lower in the combined group (2.88 ± 0.92) compared to the Laser Only group (4.53 ± 1.27), although this difference did not reach statistical significance (*p* = 0.394). These results suggest a consistent trend favoring the combined therapy in improving scar quality.

Figure 5 reveals a clearer trend: the Laser + Lipofilling group demonstrated a lower median pain score (approximately 3) and a tighter interquartile range, while the Laser Only group had higher and more dispersed scores, reaching values up to 7. These findings suggest that the combined treatment may contribute not only to structural improvement of the scar but also to better symptom control and patient comfort.

Although the limited sample size precluded statistical significance, the observed trends consistently favored the Laser + Lipofilling protocol across both objective and subjective measures. These findings support further investigation through larger, randomized controlled trials.

## 4. Discussion

The field of regenerative medicine has profoundly reshaped modern plastic and reconstructive surgery, moving from a paradigm centered on structural repair to one focused on restoring tissue function through biological modulation. Among promising tools in this context is autologous fat grafting, or lipofilling, which has evolved from a simple volumetric filler to a biologically active therapeutic agent capable of promoting scar remodeling and tissue regeneration. Adipose tissue contains not only mature adipocytes, but also a rich stromal vascular fraction (SVF) and adipose-derived stem cells (ADSCs), which secrete a plethora of cytokines and growth factors with angiogenic, immunomodulatory, and anti-fibrotic properties [11,12].

Scar formation, particularly in its hypertrophic and retractile forms, can severely compromise both the aesthetic and functional integrity of the skin. Traditional treatment modalities, including corticosteroids, radiotherapy, surgical revision, and fractional laser resurfacing, provide varying degrees of improvement but often fall short in treating deep, fibrotic, or volumetrically deficient scars. Fractional CO_2_ and Er:YAG lasers have demonstrated efficacy in scar remodeling through neocollagenesis and thermal stimulation of dermal fibroblasts, yet their effect is predominantly limited to the epidermal and superficial dermal layers [6,7,8].

These findings are in line with the extensive review by Magnani and Schweiger, who highlighted that fractional CO_2_ laser therapy is one of the most effective modalities for treating atrophic acne scars, particularly due to its ability to induce controlled dermal injury and stimulate neocollagenesis. Their review emphasized consistent clinical improvements in scar depth, texture, and overall patient satisfaction across multiple studies, especially when laser protocols were personalized based on scar morphology and skin type [17].

In recent years, hybrid fractional laser systems, combining ablative and non-ablative wavelengths, have offered a novel platform for synergistic tissue regeneration. These systems induce controlled microthermal zones that preserve surrounding structures, enhancing the safety profile and depth of penetration. When employed prior to lipofilling, the laser prepares the recipient site by stimulating vascularization, disrupting fibrotic tissue, and improving the receptivity of the stromal microenvironment to grafted adipose elements [16,18]. This concept of “preconditioning” aligns with preclinical data suggesting that injured or inflamed tissues may exhibit increased uptake and survival of mesenchymal stromal cells [14].

Our findings support this regenerative synergy. Rageh et al. demonstrated that combining autologous nanofat injection with fractional CO_2_ laser significantly improved atrophic acne scars, with enhanced skin texture and patient satisfaction compared to laser treatment alone [2].

Patients treated with the combined approach of hybrid fractional laser and autologous lipofilling experienced significantly greater improvements in scar texture, pliability, pigmentation, and symptom relief (pain, pruritus, tightness) compared to those treated with laser alone. These results are consistent with those of Roohaninasab et al., who demonstrated enhanced scar remodeling and patient satisfaction using SVF-enriched fat grafting combined with fractional laser therapy in burn scars [5]. Likewise, La Padula et al. demonstrated significant aesthetic and functional improvements in patients with severe facial burn scars treated with a combination of autologous fat grafting and fractional CO_2_ laser. Their findings revealed enhanced skin pliability, improved scar texture, and superior patient satisfaction, underscoring the synergistic regenerative effect of this dual approach [15].

Histologically, fat grafting has been shown to restore dermal architecture by increasing vascular density, normalizing collagen fiber orientation, and reducing inflammatory cell infiltration. ADSCs exert paracrine effects via secretion of VEGF, HGF, TGF-β1, and IL-10, which collectively stimulate angiogenesis, mitigate oxidative stress, and promote extracellular matrix remodeling [9,10]. Nanofat processing, as described by Tonnard et al., allows for the mechanical emulsification of lipoaspirate, preserving regenerative cells while minimizing volume, thus enabling precise intradermal delivery for superficial skin defects and fine scars [13].

The clinical versatility of fat grafting has expanded across multiple indications, from post-burn contractures and post-radiation fibrosis to acne scars and traumatic defects [19,20]. In our study, the enhanced pliability and softness of scars post-lipofilling reflect not only the volumetric restoration but also the underlying biological activity of grafted SVF elements. Particularly in atrophic and adherent scars, the dual effect of filler and regenerative action may offer an unparalleled therapeutic advantage.

Our findings should be interpreted in light of both their strengths and limitations. The relatively small and unbalanced groups, as well as the lack of randomization, reduce the statistical power of the study. Nevertheless, the inclusion of a heterogeneous scar population with different etiologies, localizations, and durations represents a strength, as it reflects real-world clinical practice. At baseline, scars had a mean duration of approximately 24 months, with etiologies including post-surgical (40%), post-traumatic (32%), and post-burn (28%), and were located on the face, extremities, and trunk. This level of detail allows a more comprehensive understanding of the patient cohort and supports the external validity of our results.

Previous studies have also highlighted the potential of combined approaches. Rageh et al. [2] and Roohaninasab et al. [5] reported significant improvements with fractional CO_2_ laser therapy, while La Padula et al. [15] demonstrated the synergistic value of combining autologous fat grafting with laser in burn sequelae. In line with these reports, our results suggest a consistent clinical trend favoring the combined protocol, even if statistical significance was not reached. Importantly, we assessed both clinician-reported outcomes (VSS) and patient-reported outcomes (VAS, satisfaction), thereby providing a broader perspective on scar remodeling and patient comfort.

Although our study reinforces existing knowledge, we believe it adds value by offering prospective evidence in a mixed cohort, demonstrating clinically meaningful trends, and integrating both objective and subjective outcome measures. Future randomized controlled trials with larger sample sizes are warranted to confirm these findings and to further refine the methodology.

Despite these promising outcomes, limitations exist. The biological variability in adipose tissue quality, differences in harvesting and processing techniques, and the absence of standardized SVF quantification protocols contribute to inter-study heterogeneity. Furthermore, although clinical improvements are evident, histological confirmation was not obtained in our cohort. Future studies should incorporate objective imaging (e.g., high-frequency ultrasound or elastography) and molecular biomarkers to assess graft survival and tissue remodeling longitudinally.

While our study focuses on the synergistic effect of laser and fat grafting for scar management, it is important to consider the broader context of energy-based devices in regenerative dermatology. For instance, radiofrequency (RF) has been shown to induce neocollagenesis and dermal remodeling through controlled heating of the dermis. In a recent systematic review, Nilforoushzadeh et al. confirmed the safety and efficacy of RF in the management of hidradenitis suppurativa, a chronic inflammatory dermatosis, suggesting potential crossover applications in post-inflammatory and scarring disorders. This opens promising avenues for combining RF with regenerative modalities such as adipose tissue derivatives, further expanding therapeutic strategies in complex cutaneous repair [21].

This study is limited by its non-randomized design, relatively small sample size, and lack of histological or imaging confirmation. Future studies should include standardized processing protocols and objective outcome measures.

In our study, follow-up was limited to 90 days, which allows the evaluation of early scar remodeling but does not capture long-term stability of results. Given that scar maturation can extend up to two years, longer-term follow-up is necessary to confirm the durability of the improvements observed. Ongoing monitoring of our cohort will provide additional data to address this issue in future publications. Moreover, we did not include objective diagnostic measurements such as high-frequency ultrasound, histopathological evaluation, or biomechanical skin analysis. While these tools could have provided additional insight into the structural basis of scar remodeling, our study was focused on clinical and patient-reported outcomes as a first-line evaluation. Future research should integrate instrumental and histological assessments to validate and complement clinical findings. Finally, another limitation is that clinical and photographic evaluations were not performed in a blinded manner, which may introduce an element of observer bias. To mitigate this, we employed validated outcome measures (VSS, VAS, patient satisfaction), but future studies should include blinded assessments by independent evaluators to further strengthen objectivity.

Our findings underscore the value of combining hybrid fractional laser technology with autologous lipofilling as a potent and biologically sound strategy in scar treatment. This integrative approach addresses both the architectural and cellular deficiencies of scarred tissue, facilitating functional and aesthetic restoration. As regenerative medicine continues to evolve, personalized protocols integrating laser therapy, ADSCs, and adjunct biomaterials may represent the future standard of care in scar management.

## 5. Conclusions

The integration of hybrid fractional laser therapy with autologous fat grafting represents a promising and biologically sound approach to scar management. Our findings demonstrate that this combined modality provides superior clinical outcomes compared to laser monotherapy, with significant improvements in pigmentation, elasticity, volume restoration, and relief from scar-related symptoms such as pain, pruritus, and tightness.

Beyond its volumetric effect, autologous fat grafting contributes biologically active elements, including adipose-derived stem cells and stromal vascular fraction, that promote angiogenesis, immunomodulation, and extracellular matrix remodeling. When applied after fractional laser-induced dermal preconditioning, fat grafts show improved integration and regenerative performance, highlighting the synergistic potential of this protocol.

This dual treatment strategy not only addresses the structural deficits of atrophic and fibrotic scars but also enhances functional and aesthetic outcomes. While further research is needed to optimize processing techniques, quantify regenerative elements, and confirm histological changes, our results support the clinical utility of laser-assisted lipofilling as an advanced tool in regenerative scar therapy.

## Figures and Tables

**Table 1:** Baseline characteristics of patients in both treatment groups. Values are expressed as mean ± SD or number of patients (%), as appropriate.

Variable	Laser Only (*n* = 40)	Laser + Lipofilling (*n* = 20)	*p*-Value
Age (mean ± SD)	42.3 ± 12.1	41.7 ± 11.8	0.84
Sex (M/F)	18/22	9/11	0.91
Scar duration (months)	13.5 ± 6.2	12.9 ± 5.9	0.72
Scar etiology (burn/post-surgical/trauma)	15/12/13	7/7/6	-
Location (face/extremities/torso)	14/17/9	7/8/5	-

**Table 2:** Evolution of Vancouver Scar Scale (VSS) and Visual Analog Scale (VAS) scores at baseline and follow-up visits (T0, T30, T60, T90). Values are reported as mean ± standard deviation.

Timepoint	VSS—Laser Only (Mean ± SD)	VSS—Laser + Lipofilling (Mean ± SD)	VAS—Laser Only	VAS—Laser + Lipofilling
T0	6.4 ± 1.1	6.3 ± 1.0	5.8 ± 1.0	5.9 ± 1.1
T30	5.5 ± 1.0	4.6 ± 0.9	4.7 ± 1.0	3.2 ± 0.8
T60	5.0 ± 1.0	3.6 ± 0.8	4.0 ± 0.9	2.1 ± 0.7
T90	4.5 ± 0.9	2.9 ± 0.7	3.6 ± 0.8	1.2 ± 0.6

**Table 3:** Clinical Features, Downtime, and Patient Satisfaction Across the Cohort.

Parameter	Laser Only (*n* = 40)	Laser + Lipofilling (*n* = 20)
**Sex**		
Male	14	7
Female	26	13
**Age (years)**		
Mean ± SD	42.1 ± 13.2	40.7 ± 11.5
Min—Max	18–65	21–63
**Scar duration (months)**		
Mean ± SD	24.5 ± 10.3	22.9 ± 9.8
Min—Max	6–60	8–48
**Scar etiology**		
Burn	12	5
Post-surgical	16	8
Post-traumatic	12	7
**Scar location**		
Face	10	6
Extremities	18	8
Torso	12	6
**Downtime (days)**		
Mean ± SD	5.2 ± 0.9	4.1 ± 0.6
Min–Max	3–7	3–5
**Satisfaction categories**		
Slight	5	1
Moderate	10	3
Significant	15	6
Marked	10	10
**Satisfaction percentage**		
Mean ± SD	58.4 ± 19.7	72.3 ± 17.5

## Abbreviations

ADSCs	Adipose-Derived Stem Cells
CO_2_	Carbon Dioxide (Laser Wavelength 10,600 nm)
Er:YAG	Erbium-doped Yttrium Aluminium Garnet
RF	Radiofrequency
SD	Standard Deviation
SVF	Stromal Vascular Fraction
VAS	Visual Analog Scale
VSS	Vancouver Scar Scale
